# A neural network model predicts community-level signaling states in a diverse microbial community

**DOI:** 10.1371/journal.pcbi.1007166

**Published:** 2019-06-24

**Authors:** Kalinga Pavan T. Silva, James Q. Boedicker

**Affiliations:** 1 Department of Physics and Astronomy, University of Southern California, Los Angeles, California, United States of America; 2 Department of Biological Sciences, University of Southern California, Los Angeles, California, United States of America; Duke University, UNITED STATES

## Abstract

Signal crosstalk within biological communication networks is common, and such crosstalk can have unexpected consequences for decision making in heterogeneous communities of cells. Here we examined crosstalk within a bacterial community composed of five strains of *Bacillus subtilis*, with each strain producing a variant of the quorum sensing peptide ComX. In isolation, each strain produced one variant of the ComX signal to induce expression of genes associated with bacterial competence. When strains were combined, a mixture of ComX variants was produced resulting in variable levels of gene expression. To examine gene regulation in mixed communities, we implemented a neural network model. Experimental quantification of asymmetric crosstalk between pairs of strains parametrized the model, enabling the accurate prediction of activity within the full five-strain network. Unlike the single strain system in which quorum sensing activated upon exceeding a threshold concentration of the signal, crosstalk within the five-strain community resulted in multiple community-level quorum sensing states, each with a unique combination of quorum sensing activation among the five strains. Quorum sensing activity of the strains within the community was influenced by the combination and ratio of strains as well as community dynamics. The community-level signaling state was altered through an external signal perturbation, and the output state depended on the timing of the perturbation. Given the ubiquity of signal crosstalk in diverse microbial communities, the application of such neural network models will increase accuracy of predicting activity within microbial consortia and enable new strategies for control and design of bacterial signaling networks.

## Author summary

Bacteria can communicate with each other using chemical signals to activate genetic expression in a process known as quorum sensing. Quorum sensing in bacteria is known to regulate a number collective behaviors in bacteria such as biofilm formation, antibiotic production and production of virulence factors which leads to bacterial infections. In a community, different species of bacteria can crosstalk using these signals, such that they regulate each other’s quorum sensing activation. Crosstalk can be either excitatory or inhibitory towards quorum sensing activation. Generally, in a bacterial community, it is not straightforward to understand how cells utilize mixtures of quorum sensing signals to regulate quorum sensing activation. To address this issue, we used a neural network approach in which we were able to predict patterns of quorum sensing activation in a diverse community of *Bacillus subtilis* cells producing five different signals and we observed that quorum sensing activation depended on signal concentration, species ratio and time sensitive external perturbations. Our findings can be useful in systematically controlling quorum sensing and potentially devising better strategies to fight bacterial infections.

## Introduction

In microbiology, quorum sensing (QS) is a process in which bacteria produce and secrete small chemical molecules known as autoinducers. Many bacteria regulate gene expression in response to the external concentration of autoinducer, including regulation of processes related to biofilm formation, virulence, and horizontal gene transfer [1–4]. Although QS is historically viewed as a process of a single species regulating its own gene expression, numerous reports have shown signal exchange between species contributed to regulation of QS phenotypes [5–10]. Such crosstalk between cells is usually the result of two bacterial strains producing chemical variants of a QS signal. QS signals have many naturally occurring chemical variants, including 56 distinct variations of acyl homoserine lactones and 231 variants of auto-inducing peptides (AIP) [11,12]. Chemically similar variants of a signal interact with QS receptors, leading to excitation or inhibition of QS activation to a variable degree [5,8,13,14]. Multiple signal inputs to a given receptor protein lead to variable levels of gene expression, making it difficult to predict community-level behaviors in the presence of two or more signaling molecules.

Signal crosstalk was first recognized when *Vibrio cholerae* and *Vibrio parahaemolyticus* produced a QS response in *Vibrio harveyi* [15]. Riedel *et al*. [16] observed QS crosstalk between *Burkholderia cepacia* and *Pseudomonas aeruginosa* where *P*. *aeruginosa* activated QS in *B*. *cepacia*. Mclean *et al*. [8] showed that a *Chromobacterium violeceum* biosensor produced different levels of QS activation when introduced with a variety of distinct Acyl-Homoserine Lactones (AHLs) separately. Geisinger *et al*. [17] detected QS activity in pairwise combinations of the Agr-I-IV QS AIP system to uncover the contribution of divergent QS alleles to variant expression of virulence determinants within *Staphylococcus aureus*. Although these studies identified the potential for crosstalk in the presence of pairwise combinations of cells, in a given environment QS crosstalk can be more complex when several QS bacteria coexist in the same community. For instance, in the human microbial gut 300–500 bacterial species are present [18] and among these populations at least ten QS species have been identified and more than eight QS signal variants have been recognized [19]. Thompson *et al*. [20] showed that the ratio between Firmicutes and Bacteroidetes can be influenced by introducing an external source of QS signals, demonstrating that changes in QS signals can have community-level influences on activity. Furthermore, in rhizosphere soil out of the 350–550 bacterial species, at least 30 species were capable of producing multiple QS signals [21], and another study found 8% of genomes in the soil contained the genes needed to activate an AHL reporter strain [22].

**Fig 1:**
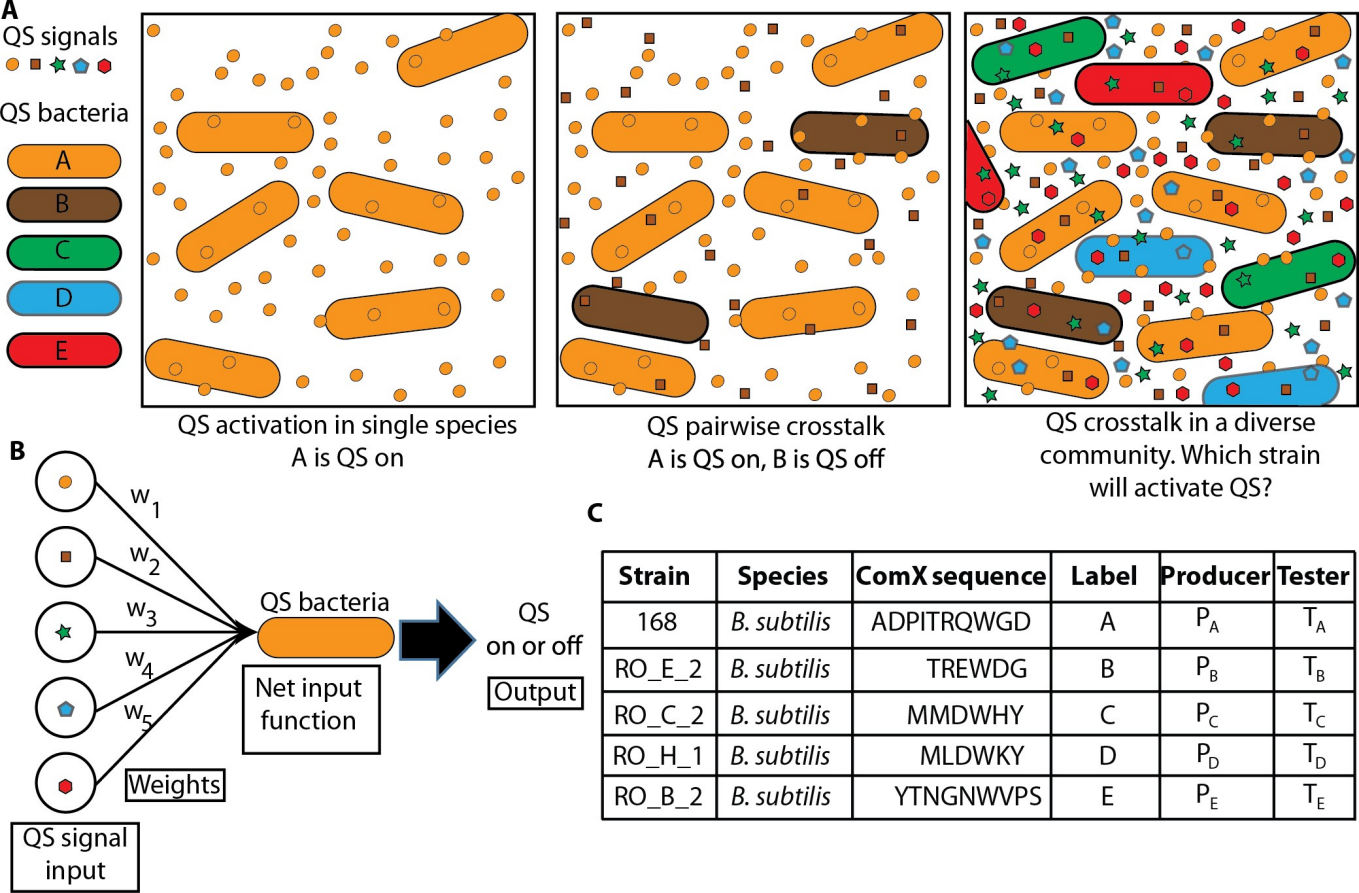
A neural network simplifies the complexity of QS crosstalk in diverse communities. A. A graphical representation of QS activation in both simple and complex communities. In isolation, individual strains activate QS. Signal crosstalk potentially changes QS activation in pairs of strains. In diverse communities, a mixture of signals determines QS activity in each strain. B. In multi-signal contexts QS activation acts as a neural network, with each strain integrating the contribution of each signal to regulate QS activity. C. Five variants of *Bacillus subtilis* produce five variants of the ComX signal [9]. These strains, labeled A-E, test the ability of the neural network model to predict QS activation in a diverse community. The producers and testers are described in the text.

Interest in engineering microbial communities to utilize multiple QS signals led to a broader characterization of gene regulation in contexts with multiple signals. Several early examples of cellular networks with multiple signals utilized the LasRI and RhlRI networks from *Pseudomonas aeruginosa*, which produce nearly orthogonal signals C4-HSL and 3-oxo-C12-HSL, with essentially zero crosstalk. Later work combined signaling networks with measurable crosstalk [23–26]. Scott *et al*. [27] conducted an extensive study on the pairwise effects of the AHLs 3-oxo-C6 HSL, 3-oxo-C8-HSL and 3-oxo-C12-HSL on the LuxR, LasR, RpaR and TraR QS systems to understand how to construct higher-level genetic circuitry for the use in microbial consortia. Wu *et al*. [7] also characterized the QS pairwise interactions of the AHLs 3-oxo-C6-HSL and 3-oxo-C12-HSL on the LuxI/R and LasI/R QS systems to better understand new directions in engineering gene networks. Our previous study investigated the robustness of signaling networks to interference by quantifying crosstalk between the LuxI/R QS system and the AHLs 3-oxo-C6-HSL and C4-HSL [5].Although these studies identified the pairwise interactions of QS in the presence of one or two auto-inducers, there is limited knowledge on how species composition, signal diversity and external perturbations would affect QS activation when more than two QS species are present, see Fig 1A. In such heterogeneous environments, with several QS signals, activation of QS in each species is interdependent, making it a challenge to predict community-wide QS activity.

Here we introduce a neural network model to examine the consequences of QS crosstalk within bacterial communities producing mixtures of QS signals, see Fig 1B. Neural network models have been commonly used to understand the network-level consequences of interactions in many complex systems, including both biological and non-biological contexts. Neural networks have been implemented in advanced analytical techniques such as deep learning, pattern recognition and image compression [28–31]. In finance and economics, neural networks are trained with historical market data to discover trends and to make successful current market predictions [32]. In the pharmaceutical industry, based on the bio activity of a large set of chemicals, neural networks are used to identify new types of drugs that can be used to treat diseases [33]. In a neural network, components have a variable state, in simplest cases active or inactive. Interactions between network components influence state dynamics and are represented as weights, with the magnitude of the weight indicating the strength of the interaction and the sign of the weight indicating whether the interaction promotes or inhibits activation. We have previously implemented a neural network to theoretically analyze the information capacity within a QS networks composed to multiple *Staphylococcus aureus* strains [34]. Here we extend these ideas, combining both experimental and theoretical results, to test whether neural network models can be used to predict and control the activation of QS in communities of bacteria producing multiple signals.

## Results

### Quantifying pairwise crosstalk between strains of *Bacillus subtilis*

**Fig 2:**
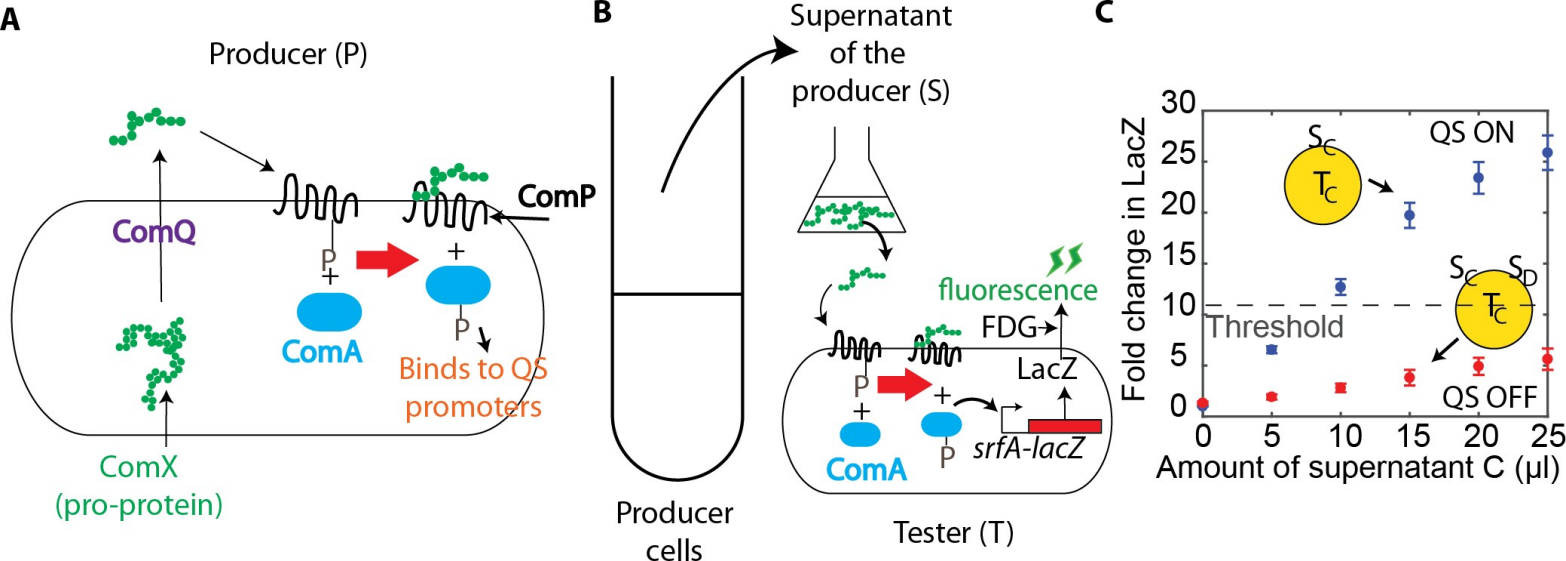
Experimental setup to measure QS activity. A. In producer cells, ComX QS signal is processed by ComQ and transported out of the cell. Binding of ComX to ComP receptor in cell membrane activates the response regulator ComA, leading to changes in the expression of QS-regulated genes. ComX, ComA, ComP are upregulated by quorum sensing activation. B. Tester cells, with a deletion of ComX and ComQ, do not produce QS signal, but increase expression of a *lacZ*, driven by QS regulated *srfA* promoter, in response to the addition of exogenous signal. The fluorogenic dye, fluorescein di-β-D-galactopyranoside (FDG), enables fluorescent readout of QS activity. C. The fold change in *lacZ* expression in tester strain C in response to addition of supernatant from strain C (in blue) and the supernatant from both strain C and 10 μl of strain D (in red). With the introduction of signal from strain D, the fold change in LacZ decreased resulting in no activation of QS. The yellow circle indicates the tester strain (T) used and the addition of either producer cells (P) or the supernatant of producer cells (S). The activation curve of individual strains defines the threshold for QS activation, with QS activated if the fold change exceeds the threshold fold change.

To test our ability to predict QS activation within a community producing multiple variants of a signal, we used five strains of *Bacillus subtilis* previously reported by Ansaldi *et al*. [9]. Each of the five strains produces a unique variant of the ComX QS signal [9,35], see Fig 1C. Each strain also had variation in the sequence of the ComP receptor protein [35,36]. In previous work [9], crosstalk between pairs of these five strains was reported, revealing a mix of both excitatory and inhibitory crosstalk between strains of variable strength. These measurements were not sufficient to construct a neural network representation of QS activity within the community, as the ratio of signals was not varied, so pairwise crosstalk between each signal was measured. We measured QS activity within mixtures of two ComX signals by combining ratios of supernatant from stationary phase culture of two “producer” strains and measuring QS activation using a “tester” strain containing a *lacZ* QS reporter (as used in the Tortosa *et al*. [35]), see Fig 2A and 2B. Producer strains produce the ComX signals and self-activate QS while the tester strains do not produce the ComX signal, but can activate QS if there is an external supply of ComX. QS activity was measured with a fluorogenic LacZ assay in a 96 well plate reader, see Fig A in S1 Text and methods for tester responses with cognate signal. The fold change in LacZ expression is the ratio of LacZ expression in the presence and absence of ComX signal, see methods for further details. In individual strains, QS activity increased and approached saturation, Fig 2C and Fig B in S1 Text. The yellow circles shown in Fig 2C are a representation used throughout the manuscript to symbolize the addition of either a producer cells (P) or a supernatant from producer cells (S) to a tester (T). The tester is indicated in the middle of the circle while the producer or supernatant strains are indicated on the circumference. Each producer supernatants was extracted only once and stored at -20 ^o^C. For all experiments the same batch of supernatant was used such that signal concentrations were consistent for all measurements, see methods for further details. The activity of the supernatant remained consistent throughout the study, see Fig C in S1 Text.

Next we examined QS activity upon exposure to mixtures of signal. As shown in Fig 2C, in the presence of second signal D, the QS activity in strain C is inhibited. To quantify the interaction weight between strains, we constructed a mathematical model. The model is based on a set of differential equations and accounts for signal crosstalk by introducing a crosstalk weight for each pair of receptor and signal, similar to models used previously [5,34]. Specifically, the expression of the QS-regulated gene *lacZ* follows:

$$\frac{\partial L}{\partial t}=\rho_{L}\;n_{i}\;\left(f_{i}(\frac{{C_{eff,i}}^{m}}{{C_{eff,i}}^{m}+{\theta_{i}}^{m}})+1\right)-\gamma_{L}L,$$ (1)

where the effective concentration of signal as the result of crosstalk is,

$$C_{eff,i}=\sum_{j}w_{i,j}c_{j}.$$ (2)

In a population of *n*_*i*_ cells, *lacZ* expression occurs at a basal rate of *ρ*_*L*_ and upon QS activation the production rate is increased by a fold change *f*_*i*_. A Hill’s function is used to represent the scaling of QS-activity with signal concentration, with a Hill coefficient of *m* and the concentration of half maximum of *θ*_*i*_. LacZ will degrade at a rate of *γ*_*L*_. An effective concentration, *C*_*eff*,*i*_ is used to account for the excitatory or inhibitory influence of each signal on QS activation in the i^th^ strain. The interaction weight *w*_*i*,*j*_ accounts for the magnitude and sign of the interaction between a ComX signal from *B*. *subtilis* strain *j* on QS activation in *B*. *subtilis* strain *i*. As in a neural network mode, the sum of these weighted interactions predicts the activity of each node (strain) for mixtures of inputs (signals). The self-weight (*w*_*i*,*i*_) is one for all strains, and if *C*_*eff*_<0, we assume that *C*_*eff*_ = 0.

Similar to previous studies [5,37–39], the i^th^ tester grows at a rate μ in a volume v through a logistic growth equation,

$$\frac{\partial n_{i}}{\partial t}=\mu_{i}\;n_{i}\;(1-\frac{n_{total}}{s\;v}).$$ (3)

Here *n*_*i*_ is the amount of the i^th^ tester cell, *μ*_*i*_ is the growth rate of the cells (given in Fig D in S1 Text), *n*_*tota*l_ is the total number of cells in the system, and *s* is the maximum density of cells reached by the culture. In simulations, tester strains in the well of a plate grew from a cell density of 10^8^ to 10^9^ cell per mL. Over time, QS-regulated *lacZ* was produced following Eqs 1, 2 and 3. LacZ concentrations in the culture were simulated for tester cells exposed to no signal as well as for a specified mixture of signal to calculate the fold change in *lacZ* expression, see methods section and Table A in S1 Text for model parameters [37,40,41]. We first simulated the response of the testers when mixed only with the cognate signal supernatant and these simulations were used to fit experimental data to obtain *f*_*i*_ and *θ*_*i*_ for each strain, further details given in the methods section, see Figs E and F in S1 Text and Table 1. The best fit *f*_*i*_ and *θ*_*i*_ values were used to generate the simulation curve, and the fold change in LacZ of this simulated curve at *θ*_*i*_ was defined as the threshold value of LacZ fold change needed for QS activation, see Fig 2C and Fig F in S1 Text. In the methods section we describe how to convert from supernatant volume to a relative signal concentration. This is an approximation of the signal concentration in the supernatant and does not take into account variability of signal production for each strain. The five strains had five distinct threshold values needed for QS activation, see Fig F in S1 Text.

**Table 1:** The best fit values for *f*_*i*_ and *θ*_*i*_. These values were extracted by minimizing the root-mean squared error between the experimental and simulated data points of the LacZ fold changes vs. amount of supernatant.

Strain (i)	*f*_*i*_	*θ*_*i*_ (nM)
A	5.955 ± 0.002	1.393 ± 0.002
B	4.083 ± 0.001	1.633 ± 0.004
C	24.839 ± 0.004	1.364 ± 0.002
D	8.345 ± 0.005	1.394 ± 0.001
E	6.951 ± 0.002	1.815 ± 0.003

**Fig 3:**
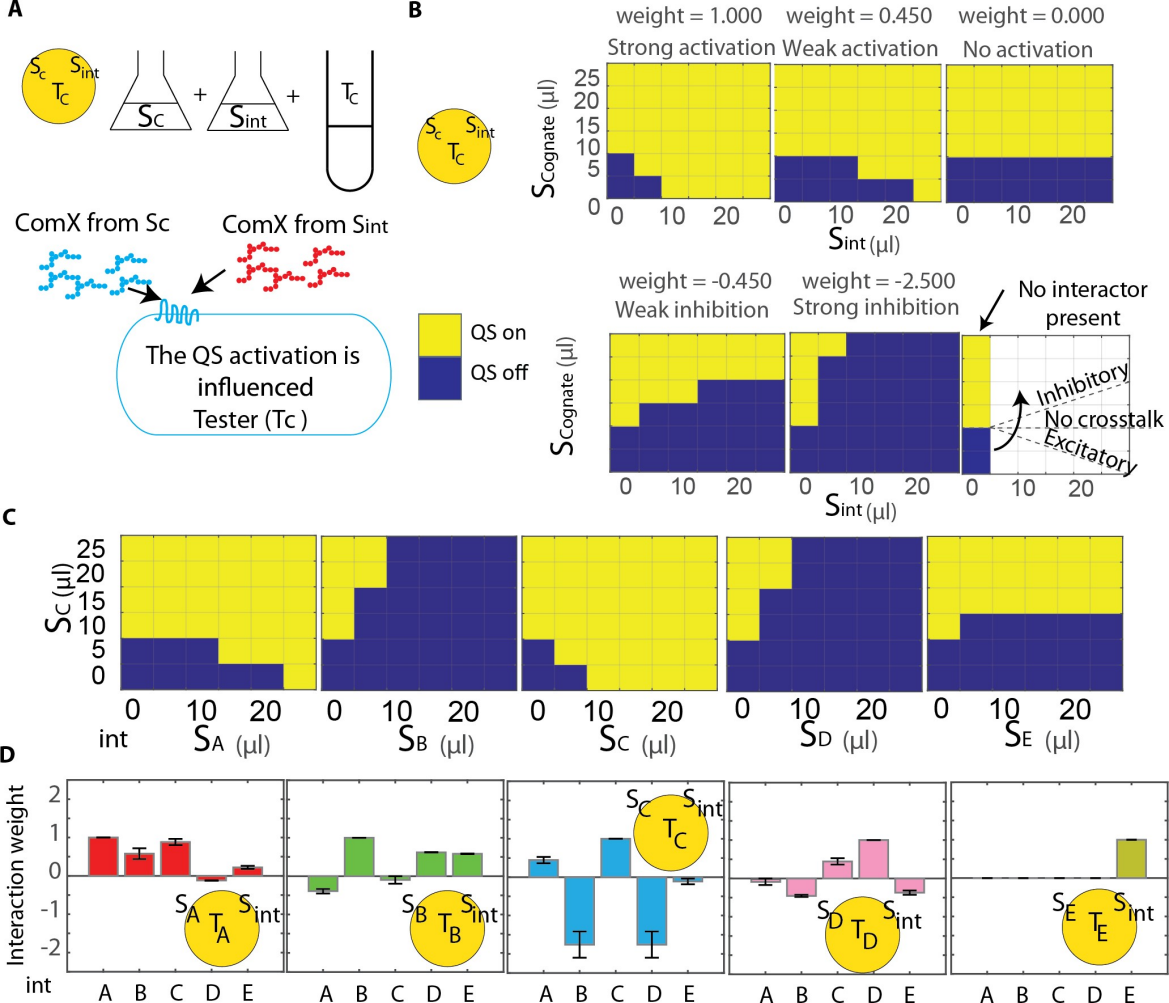
Pairwise measurements. A. QS activation in the presence of two signals is measured by combining supernatant containing the cognate signal (S_c_) with supernatant containing the interacting signal (S_int_) with a tester strain. B. Simulation results show the activation landscape depended on the crosstalk weight of the interacting signal. Results are shown for strain C, where *f* = 24.839 and *θ* = 1.364 nM. Yellow and blue boxes indicate combinations of signals for which QS did or did not activate, respectively. QS activation is defined as the fold change in *lacZ* expression associated with a cognate signal concentration of *θ*. C. Experimentally measured activation landscapes for strain C. D. Experimental measurements were used to extract the crosstalk weight for each pair of signal and receptor. The error bars represent the range of weight values which gives the same activation landscape, see Figs G-L in in S1 Text.

Simulations were done to obtain the response of the testers when the cognate signal was mixed with an interacting signal, see Fig 3A. In Fig 3B, we have simulated a simplified representation with QS on (yellow boxes) and off (blue boxes), for such a case, and we observe that this pattern of activity (or QS activation landscape) changes depending on the weight of the interacting signal. QS on and off were determined by testing whether the QS activity for a given combination of cognate and interacting signal would be higher or lower than the threshold calculated previously. We obtained similar activation landscapes for experiments considering the same thresholds for each strain, Fig 3C and Figs G-K in S1 Text. Fig 3C shows the activation landscape of T_C._ For each strain, between 0 and 25 μL of supernatant from each producer strain was mixed with 0 to 25 μL of supernatant from a second strain. 25 μL was chosen as the maximum volume of supernatant as individual strains required 10 μL or 15 μL to activate QS, giving sufficient dynamic range to measure even strong inhibition. We compared the experimental activation landscapes with the simulated activation landscapes to extract the weights of each *B*. *subtilis* tester and the corresponding interacting strain. For example, simulations for the response of strain C to mixtures of signal from strains A and C indicate w_C,A_ between 0.363 and 0.527 reproduce the experimental measurements of quorum sensing activation. Therefore, w_C,A_ is reported as the mean of these values with error bars indicating the range of possible values. Using this method, the interaction weight was calculated for each pair of strains, see Fig 3D.

The weights calculated for each strain revealed a rich network of signaling interactions within the 5 strain community, Fig 3D and Fig L in S1 Text. For example QS activation in strain C was activated by signal from strains A or C, strongly inhibited by signal from strains B or D, and only weakly responded to signal from strain E. Strain E on the other hand only responded to its own signal and was not influenced by the signal from any other strain tested. Note that these simulations predict QS activation to a static input of each signal. Later we discuss how quorum sensing activity depends on the dynamics of signal production and cell growth.

### Pairwise weights predict quorum sensing activation patterns in the 5-strain community

**Fig 4:**
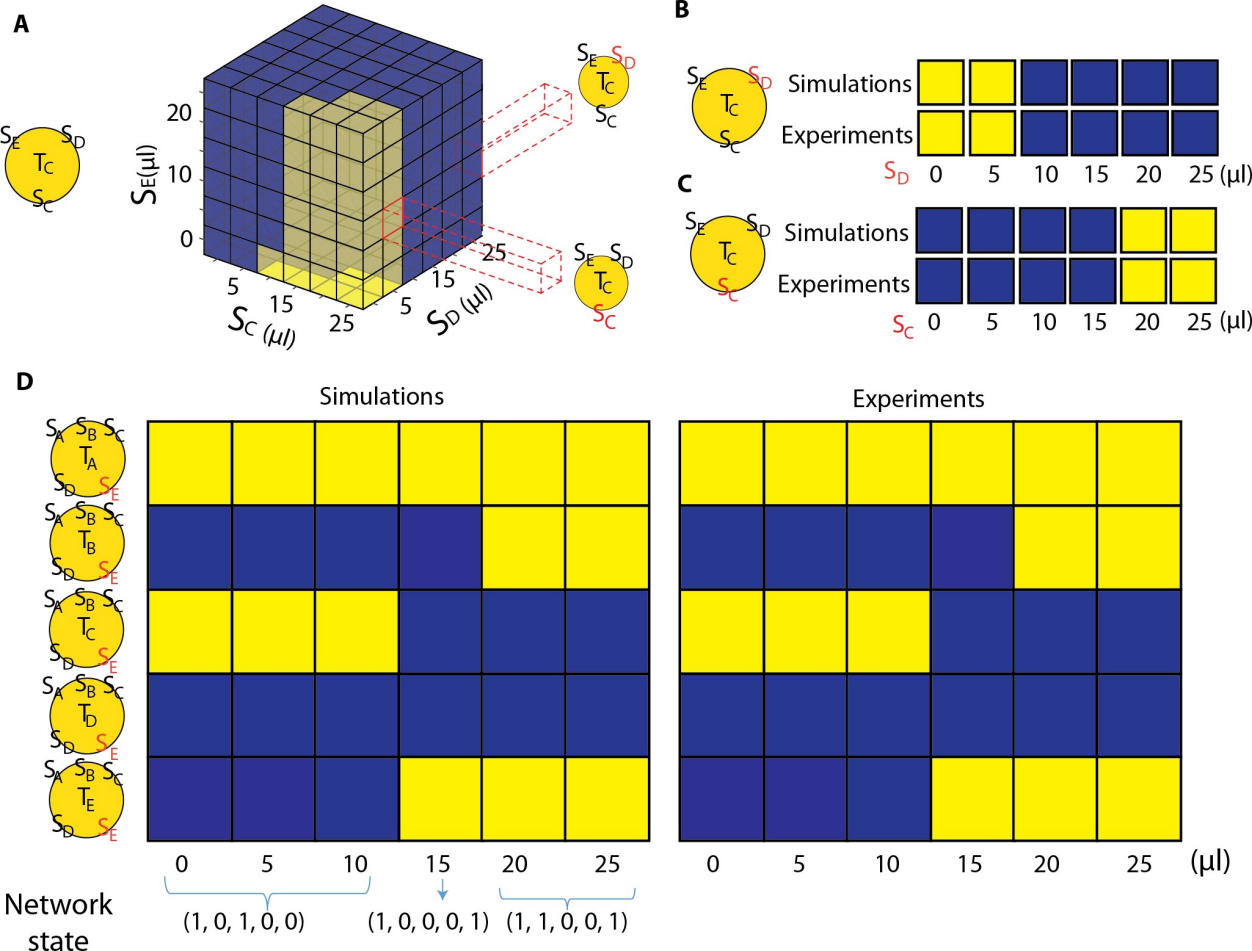
Experimental validations of the model predictions when more than two signals are present. A. Simulations using the neural network model predicted the activity of a tester strain in the presence of three ComX signals. The response of tester T_c_ was simulated in the presence of S_C_, S_E_ and S_D_. The cube of colored boxes shows how the tester strain C will respond to different combinations of signal, blue boxes represent QS off and yellow boxes represent QS on. The dashed lines show slices of the cube that were tested experimentally. Experimental validation of theoretical predictions of QS activation in the presence of 3 signals. One signal concentration was varied as the other two ComX supernatants were kept at a constant value. For, B. S_C_ = 25 μl and S_E_ = 10 μl and S_D_ was changed from 0–25 μl. C. S_E_ = 10 μl and S_D_ = 5 μl and S_C_ was changed from 0–25 μl. D. In the presence of all five ComX signal supernatants, we observed a range of activation patterns for the community. The community-level signaling state, represented by a binary string of numbers indicating the QS activity of each strain, changes depending on the ratio of signals. S_A_, S_B_, S_C_ and S_D_ were held constant at 10 μl, 4 μl, 15 μl and 1 μl respectively while S_E_ was varied from 0 to 25 μl, in 5 μl increments. Experimental measurements of QS activity in the tester strains matched theoretical predictions. In the yellow circles, the red text indicates the signal concentration that was varied.

The extraction of the pairwise interaction weights in the previous section enabled us to apply a neural network model to predict QS activation patterns in groups of 3 or more strains. This model is a fully connected, single-layer network without any hidden layers. In the model the nodes represent each strain in the community with the weighted connections representing signal exchange between each strain. In Fig 4A, we have predicted the response of T_C_ in the presence of S_C_, S_D_ and S_E_. The interaction weights are not retrained with each new set of experimental data, instead the interaction weights calculated in Fig 3 are inherited for all subsequent model predictions. Hence in Fig 4A, we use the weights w_C,C_, w_C,D_, w_C,E_ to simulate the response of T_c_. As shown in Fig 4B and 4C, model predictions were verified in experimental measurements. In experiments, supernatants from three different strains were mixed at a specific ratio with a tester strain, and the expression of QS genes was measured using the fluorogenic LacZ indicator. Each measurement used only a single tester strain, and separate experiments were carried out in parallel to determine the response of the full community. Experimental measurements reproduced the predicted pattern of QS activation as ratios of supernatant were varied. This shows that a simple one layered network is capable of predicting the QS output in the presence of three signals. Analysis of QS activation in the presence of three signals revealed the concept of a community-level signaling state. In a microbial community producing multiple signal variants, crosstalk between these signaling systems potentially leads to the activation of QS in subsets of the community. The exact combination and ratio of signals, as well as the structure of the crosstalk network will determine which strains activate QS.

The neural network model, with the pairwise weights, predicted the community-level signaling state and its sensitivity to changes in signal concentrations, as shown in Fig 4D and Fig M in S1 Text. The signaling state of the 5-strain community is shown as the amount of signal from strain E was varied. The community-level QS state can be represented as a binary string, with a 1 or 0 in each position of the string indicating whether QS will activate or not activate, respectively, for each strain. For example, at 0 μL of supernatant from strain E, the predicted string was (1,0,1,0,0), which indicated that only strains A and C would activate QS under these conditions. As the amount of strain E supernatant was increased, the community-level state changed twice, first to (1,0,0,0,1) and then to (1,1,0,0,1). These predictions from the model were borne out in experimental measurements, as the two transitions of the community-level signaling state were observed as the volume of supernatant from strain E increased.

### The network state depends on the inoculation ratio of strains

**Fig 5:**
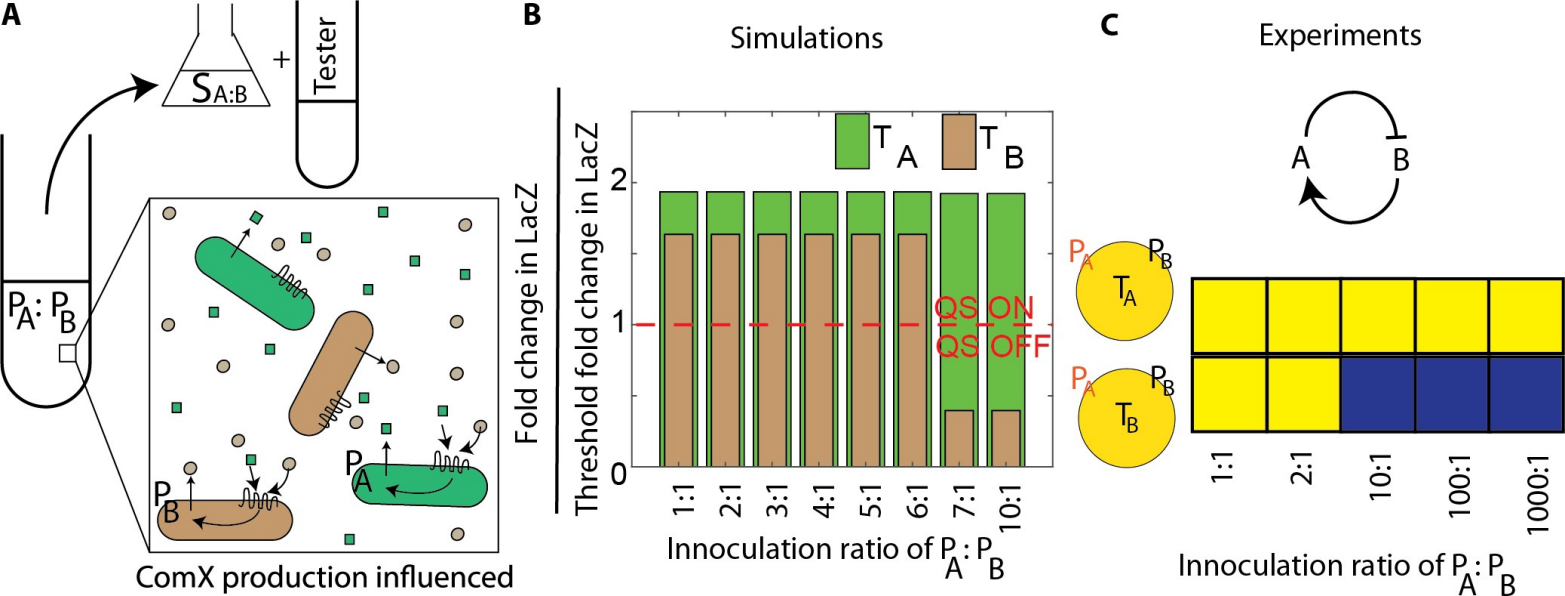
Initial loading ratio of the producers dictated the final QS activation state. A. Producers A and B were mixed together at a specified ratio and grown to stationary phase. Supernatant of the coculture was applied to tester strains to determine the QS activity of each strain. B. Simulations predicted the fold change in LacZ normalized to the threshold level of LacZ expression defined as QS activation. Strain A activated QS at all ratios of cells, whereas QS activation of strain B occurred only at specific inoculation ratios of the two strains. C. Experimental validation of the simulation results. Supernatant from the coculture of producers grown 2 hours past exponential phase was used to determine QS activity. The arrow map shows the nature of crosstalk between A and B, with A inhibiting B and B activating A.

In the previous sections, we determined that a neural network model could predict the consequences of QS crosstalk within a 5-strain community of *B*. *subtilis*. These predictions were tested in experiments in which specified ratios of supernatant from stationary phase cultures were combined with tester strains to measure QS activity. Next we wanted to verify that the model could also predict QS activity for mixtures of strains growing from low density culture, see Fig 5A. These experiments revealed how inoculation ratios influenced QS activation in a multi-strain community.

As cells grow, release signals, and potentially activate QS, the signal concentrations will change over time until reaching a steady state [5,37,39]. To simulate signal production, cell growth, and the expression of QS-regulated *lacZ*, Eqs 1–4 were used. Eq 4 describes the change in signal concentration (*c*_*i*_) over time for the i^th^ strain as the result of signal production and degradation. The signal production of the i^th^ strain will be influenced by all the other strains that are present, as captured by *c*_*eff*,*i*_ defined in Eq 2.

$$\frac{\partial c_{i}}{\partial t}=\rho_{c_{i}}\;n_{i}\;\left(f_{i}\;(\frac{{c_{eff,i}}^{m}}{{c_{eff,i}}^{m}+{\theta_{i}}^{m}})+1\right)-\gamma_{c_{i}}c_{i}.$$ (4)

In a population of *n*_*i*_ cells of the i^th^ strain, signal production occurs at a basal rate of $\rho_{c_{i}}$ and at QS activation, this rate is increased by a fold change *f*_*i*_. A Hill’s function is used to represent the scaling of signal production with effective signal concentration *c*_*eff*,*i*_, with a Hill coefficient of *m* and the concentration of half maximum of *θ*_*i*_. *c*_*eff*,*i*_ incorporates the QS crosstalk as mentioned previously. The signals will degrade at a rate of $\gamma_{c_{i}}$. For this case, in Eq 3, $n_{total}=\sum_{j=1}^{k}n_{j}$, where k is the total amount of producer strains mixed together. Parameter values are listed in Table A in S1 Text.

In simulations, using Eqs 3 and 4, P_A_ and P_B_ were mixed at different ratios and the concentration profile of the ComX signals for both were plotted with respect to time, see Fig N in S1 Text and methods for further details. Fig 5B shows how the inoculation ratio of P_A_ to P_B_ influenced expression of the QS-regulated *lacZ* gene. The simulated fold change in LacZ was normalized by the threshold fold change in LacZ needed for QS activation. Here the fold change in LacZ is determined by applying the supernatant of the mixture P_A_ and P_B_ after 10 hours growth to the testers T_A_ and T_B_ separately. We observed that above a 6:1 inoculation ratio of P_A_ to P_B_, strain B does not produce a sufficient concentration for QS activation, whereas strain A activated QS at all strain ratios. In experiments, we mixed P_A_ and P_B_ at ratios of 1:1, 2:1, 10:1, 100:1, 1000:1, and grew them for 10 hours, see Fig 5C. Supernatants of these mixtures activated QS in T_A_ at all ratios, whereas T_B_ was only activated at 1:1 and 2:1 ratios. P_A_ and P_B_ as both are based on the *B*. *subtilis* 168 strain such that any other competition is minimized. As seen in Fig 5A, mixing these strains would impact the ComX production of each other. These results demonstrate that the inoculation ratio of strains dictated the final QS state of the community.

### Using ComX perturbations to switch community-level QS activation states

We have shown that the QS activation of species depends on species composition, ratio of the number of species, signal composition and signal concentration. Next we tested if the QS activity of a multi-strain community could be influenced by a perturbation of added signal, and whether the timing of signal addition impacted the response to the perturbation.

**Fig 6:**
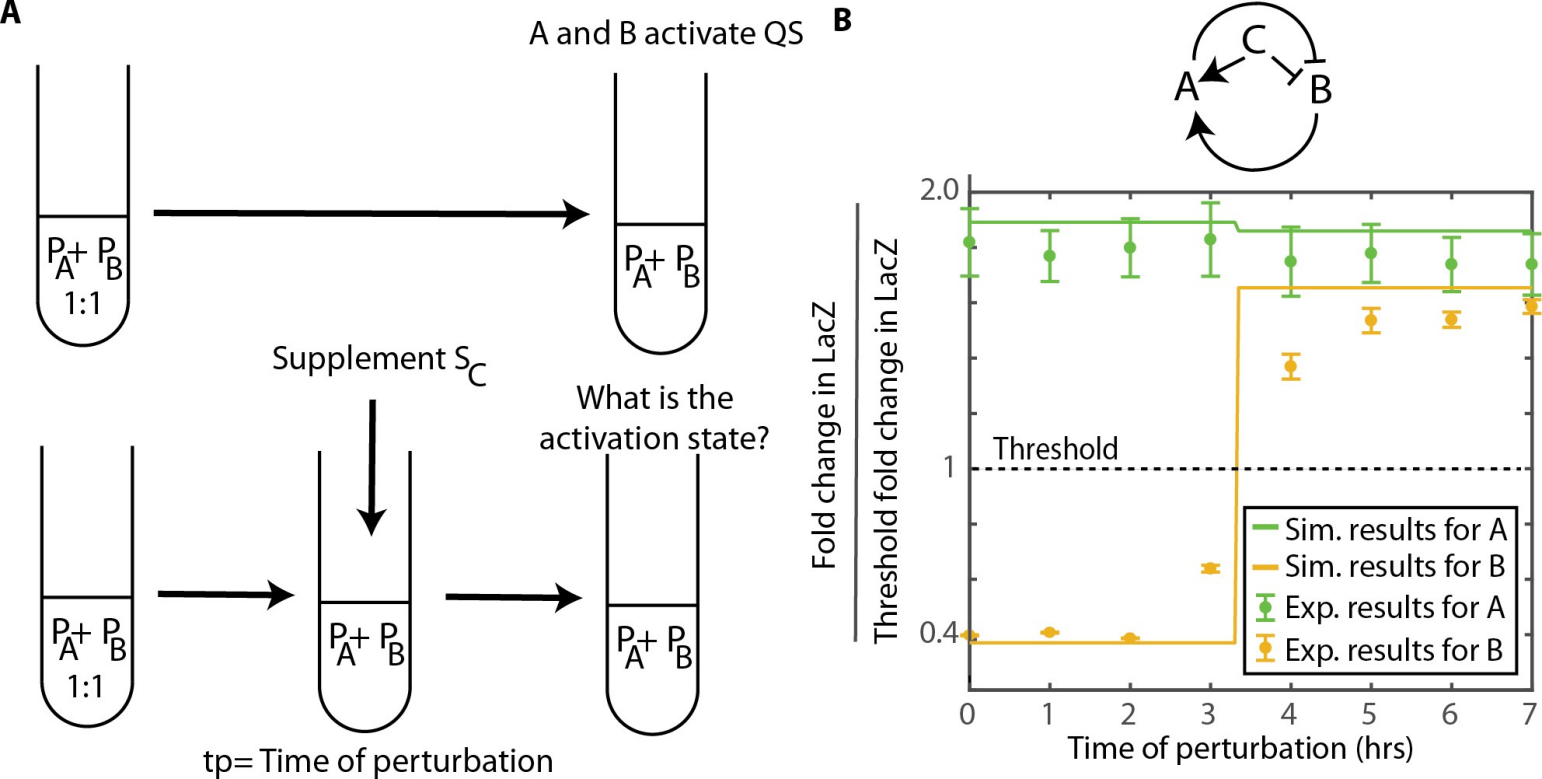
Perturbing the system to change the QS activation state. A. When P_A_: P_B_ are mixed at a ratio of 1:1, both A and B will activate QS. A perturbation of signal from strain C was introduced to the system after t_p_ hours. B. The map shows the nature of crosstalk between strains. Here, we represent the fold change in LacZ normalized to the threshold fold change needed for QS activation for both T_A_ and T_B_ vs. the time at which the perturbation was introduced. The lines represent the simulation results and the data points are from experiments. The error bars represent the standard error from 3 sets of replicates. We observe that the change in the activation state of T_B_ depends on the time at which the perturbation was introduced.

As depicted in Fig 6A, in both simulation and experiments we mixed together producers A and B at a ratio of 1:1, and measure the QS activity of tester strains A and B. In the control experiment, the coculture was not perturbed, and we expected results as in Fig 5, strains A and B both activate QS. Duplicate cocultures were perturbed by the addition of 200 μl of supernatant from strain C at various points of time after inoculation. As shown in Fig 6B, both simulations and experiments confirm that the addition of signal C had the potential to alter the activation of QS in the coculture, see Fig O in S1 Text. Signal C inhibits QS activation in strain B and promotes QS activation in strain A. However, whether the community-level signaling state changed from (1,0) to (1,1) depended on the time of the perturbation. The simulations showed either QS on or QS off for T_B_, since after 10 hours the signal concentration reached a steady-state that would either be above or below the threshold for QS activation, see Fig O in S1 Text. Addition of supernatant from strain C prior to 4 hours resulted in strain B not activating QS, whereas a perturbation at 4 hours or later did not alter QS activation of either strain. As above, the QS activity for these cases was tested with supernatants extracted from cultures that were grown 2 hours past exponential phase.

## Discussion

In this study, we analyzed QS activation within a community of five different *B*. *subtilis* strains that produce distinct ComX signals. The pairwise interactions measured within the 5-strain community were consistent with crosstalk patterns reported in Tortosa *et al*. and Ansaldi *et al*. [9,35], Fig P in S1 Text. Although crosstalk between pairs of QS systems have been observed previously [5,6,9,35,37,42], here for the first time we predict the consequences of signal crosstalk on QS activity in a community utilizing multiple signal variants. In natural environments, QS crosstalk is prominent and often regulates genes associated with virulence and biofilm formation [14,43–47]. Many natural communities produce diverse sets of signaling molecules [48,49], resulting in entangled and interdependent gene expression within the community. For instance, in a recent report [50], it was suggested that QS in B. subtilis can regulate ComX degradative enzymes, which in turn will inhibit QS activation. Therefore, QS crosstalk could potentially be entangled with such ComX degradation. Neural networks, inspired by the decision making within densely interconnected neurons, enable predictions of activity within complex systems with many interacting components. Here we demonstrate the consequences of signal crosstalk on community-level gene expression states can be accurately predicted using a neural network model with pairwise interactions.

Many previous studies on QS crosstalk have implied that QS activation state is solely dependent on the type, or in our case sign, of the crosstalk [7,45]. In the presence of all five ComX signals, we observed that the QS activation state of one strain will be different depending on the exact mixture of ComX concentrations. Therefore simply knowing the community composition will not give an accurate picture of the potential for QS activation. Communities with identical membership can be driven towards multiple community-level signaling states; ratios of strains and the structure of the interaction network determine which strains or species activate QS. The history of the community also dictates activation patterns, as the temporal dynamics of signal accumulation, as influenced by strain inoculation ratios and perturbations, dictate QS activation states. The neural network model may enable the design of perturbations to redirect QS activation within bacterial communities. As QS activation is known to influence the real-world problems such as infections and biofouling, this predictive model of community-level activity should be relevant for industrial and medical applications [45,51,52].

Although the utility of a neural network model of QS was demonstrated for only one class of QS signals, cyclic autoinducing peptides used by several species of Gram-positive bacteria, the model should be easily extended to communities using the acyl-homoserine lactone signals common in Gram-negative QS. Crosstalk as the result of two cells producing chemically related signaling molecules is not unique to cyclic autoinducing peptides [7,53]. Crosstalk within AHL networks or other signaling networks may have a different distribution of weights, which would impact the number and sensitivity of community-level signaling states. Here we assumed each signal accumulates to the same concentration in the supernatant, however each strain might accumulate different concentrations of signal. Variability in signal concentration in the supernatant therefore modulated each interaction weight by an unknown multiplication factor, a factor which could be determined through measurements of signal concentrations.

In addition, although the 5-strain *B*. *subtilis* community studied here was densely connected, i.e. each strain was connected to every other strain in the network, and the modelling framework could be applied to networks in which only subsets of strains participate in quorum sensing crosstalk. The model might also be relevant to other types of microbial interaction [54]. Some bacterial species detect more than one signal, such as strains which utilize AI-2 and AHLs or for example *V*. *cholerae* which combines information from four chemically distinct signals [55]. Mapping these situations onto the neural network model should give deeper insights into QS regulation in diverse microbial communities and reveal how the structure of the network determines the community’s ability to exchange information and coordinate group activity.

## Materials and methods

### Bacterial strains, growth media and conditions

All strains used in this study were obtained from the study Ansaldi *et al*. [9]. The producer cells produced the ComX pro-protein which is modified and processed by ComQ, in turn, releasing the ComX pheromone to the extracellular environment. Released ComX pheromones bind to the ComP membrane protein to phosphorylate ComA and activate QS. The testers cannot produce the ComX signal due to the disruption of the *comQ* and *comX* genes but can activate QS when the signal is exogenously added to produce LacZ under the QS-regulated *srfA* promoter.

All the testers and producers were isogenic apart from producing distinct pheromones and receptors. To construct the strains labeled from B-E, the *comQXP* genes of the *Bacillus subtilis* 168 were replaced with the foreign *comQXP* genes from four distinct *Bacillus* species [9,35]. The *Bacillus subtilis* strains were grown in the *Bacillus* competence media as described previously [56]. This media contained (w/v) 1.00% sodium Lactate, 0.25% yeast extract, 0.20% ammonium sulfate, 1.40% dipotassium phosphate, 0.60%; mono potassium phosphate, 0.10% sodium citrate-2H_2_0, 0.02% magnesium sulfate*7H_2_0 and 0.40% of glucose. All cultures were grown at 37°C and 200 RPM in competence media.

Tester strains grown overnight, diluted 1/1000 in fresh media, and grown for an additional 10 hours before measurements. To extract supernatant, producers were grown for 10 hours after 1/1000 dilution of overnight culture. Cultures were centrifuged for 4 min at 4000 RPM to collect supernatant. The supernatant was passed through a 0.2 μm VWR syringe filter. Filtered supernatants were stored at -20°C. A single batch of supernatant from each strain was used for all experiments.

When mixing the producers strains together producers were grown in separate cultures for 10 hours, diluted 1/1000 in fresh competence media and grown for an additional 3 hours. Growth for 3 hours at low density was to ensure QS was not active prior to mixing strains together. After 3 hours of growth, cultures reached a final OD of 0.2–0.3. Cultures of producer strains were mixed together at different ratios. For example, in the 1:1000 case, we mixed 1 μl of P_A_ with 999 μl of P_B_ together into 3 mL of competence media. Mixed cultures were grown for 10 hours. Since all producers and testers were based on the *B*. *subtilis* 168 and they were near isogenic, no growth interactions occurred between strains when mixed together. To measure activity, supernatant was extracted from producer cocultures as described above. In perturbation experiments, supernatant from strain C was added to the culture from 0 and 7 hours at every 1 hour interval, after mixing producer strains together.

### β-galactosidase assay

For the indicator, we used fluorescein di-β-D-galactopyranoside (FDG) [35,57], and the fluorescence was detected by using a Tecan plate reader with a 96 well plate. In the 96 well plate, we mixed 25 μl of the testers, 25 μl of the FDG at 0.04 mg ml^-1^ (FDG in the competence media), and 75 μl of fresh competence media. The remainder of the 200 μl volume was a variable combination of filtered supernatants and spent media. For consistency, all wells had a total of 75 μl of spent media, a combination of supernatant from producer strains and supernatant from the tester strain (which did not contain any signal). Testers were loaded after 10 hours growth, as described above. Fluorescence was detected with an excitation wavelength of 480 nm and an emission wavelength of 514 nm. For the mixed producer experiments, we added 150 μl of supernatant with the testers and FDG. The absorbance was detected at 600 nm to enable calculation of fluorescence per cell, as described previously [37]. Similar to previous studies [9,35], the LacZ expression level was calculated by obtaining the rate of fluorescence increase, which is the gradient of the fluorescence per cell vs time in the linear region between 0–5 hrs, see Fig A in S1 Text. The fold change in LacZ was calculated by taking the ratio of a given LacZ expression level with the LacZ expression level when no signal is present.

### Growth measurements

To obtain the growth rates, the cells were grown in the *Bacillus* competence media, as mentioned above. We then diluted the cells 1000 fold in 3 mL of competence media and grew them at 37°C and 200 RPM. Dilutions of the culture were plated on competence media agar plates at every 1 hour interval during growth for 7 hrs, and these plates were incubated for 16 hrs at 37 ^0^C. For each time point 2 sets of replicates were considered, and after 10 hours growth, the number of colonies were counted to get the colony forming units. The growth rates were obtained from a fit to the linear region in the growth curve.

### Mathematical modelling and simulations

To perform the simulations described in the manuscript we used MATLAB 2016b. We used the finite difference method to simulate the change in LacZ expression with respect to time, using a time step of 1 min. We calculated the LacZ production rate by simulating the increase in LacZ using Eq 1.

For each strain i, as shown in Table 1, *f*_*i*_ and *θ*_*i*_ were distinct. We obtained *f*_*i*_ and *θ*_*i*_ by minimizing the root-mean squared error and determining the best fit curve between the experimental data points and simulation curve, see Figs E and F in S1 Text and Table 1. The thresholds to determine QS activation for each strain were different based on the unique values for *f*_*i*_ and *θ*_*i*_, see Fig F in S1 Text. To plot the QS activation patterns of each tester for the pair-wise case, using unique *f*_*i*_ and *θ*_*i*_, values we simulated the fold change in LacZ for each combination of signals and tested if the fold change in LacZ was above or below the threshold of the tester. If it was above, we assigned the yellow color and if it was below, we assigned a blue color. The activation pattern of the simulations, as seen in Fig 3A, was compared with the experimental patterns to extract the weights. We observe the same activation pattern for a narrow range of weights, see Figs G-L in S1 Text.

To convert the loading volume of the supernatant to the signal concentration we considered the following method. Previous studies have reported the final concentration of ComX in media after 2 hours growth from late exponential phase is approximately 30 nM [58,59]. Since a volume of x μl of the supernatant at 30 nM gets diluted in a total of 200 μl in the 96 well plates, see methods for further details, we can calculate the signal concentration = 30 nM (x/200).

For the simulations with the P_A_ and P_B_ grown together, we considered an initial density of 10^6^ cells/ml. This was based on the dilutions used in the experiments. We mixed P_A_ and P_B_ at different ratios and simulated the change in ComX concentrations over time. In mixed cultures of P_A_ and P_B_, the production of ComX concentration depends on the crosstalk between the two producer strains, as signal production is regulated by quorum sensing. The crosstalk is taken into account by calculating C_eff,i_ using Eq 2. Using Eqs 1–4 and a finite difference method with a time step of 1 min, we simulated the change in cell number and signal concentration over time to determine if the mixture of signals that accumulated after 10 hrs would activate quorum sensing in strains A or B. A similar procedure was followed to simulate the effect of a signal perturbation in Fig 6. Perturbations were introduced between 0 to 7 hrs, and the response of the tester strains to the signal mixture that accumulated after 10 hrs was predicted.

## Supporting information

S1 Text Supporting figures and table. S1 Text contains 16 supporting figures and a table of parameter values used in simulations.
(DOCX)

S1 Dataset Data associated with figures from the main text. (ZIP)
