# Supplementary material for: A neural network model predicts community-level signaling states in a diverse microbial community
Source: PLoS Comput Biol. 2019 Jun 24;15(6):e1007166. doi: 10.1371/journal.pcbi.1007166 (PMC6611639; doi:10.1371/journal.pcbi.1007166)
Supplement: S1 Text — contains 16 supporting figures and a table of parameter values used in simulations. (DOCX) [file pcbi.1007166.s001.docx]

**Supporting Information: A neural network model predicts community-level signaling states in a diverse microbial community**

**Kalinga Pavan T. Silva, James Q. Boedicker**

Fig. A: Fluorescence per cell vs time for the testers.

Fig. B: The fold changes in the LacZ expression.

Fig. C: Consistency of the response of the testers.

Fig. D: Doubling times for the producers and testers.

Table A: Parameter values used in the study for the modeling.

Fig. E: The root mean squared error minimized.

Fig. F: The best fit curves for f and Ɵ.

Fig. G: The quorum sensing activation landscape for the tester A.

Fig. H: The quorum sensing activation landscape for the tester B.

Fig. I: The quorum sensing activation landscape for the tester C.

Fig. J: The quorum sensing activation landscape for the tester D.

Fig. K: The quorum sensing activation landscape for the tester E.

Fig. L: The overall pairwise quorum sensing interaction map.

Fig. M: LacZ fold change comparisons of the 5 supernatant case from simulations and experiments.

Fig. N: Simulation results of the ComX concentration normalized by Ɵ over time.

Fig. O: Simulation results of the ComX concentration normalized by Ɵ over time when the system is perturbed.

Fig. P: The comparisons of the pairwise crosstalk of our results with Ansaldi *et al.*


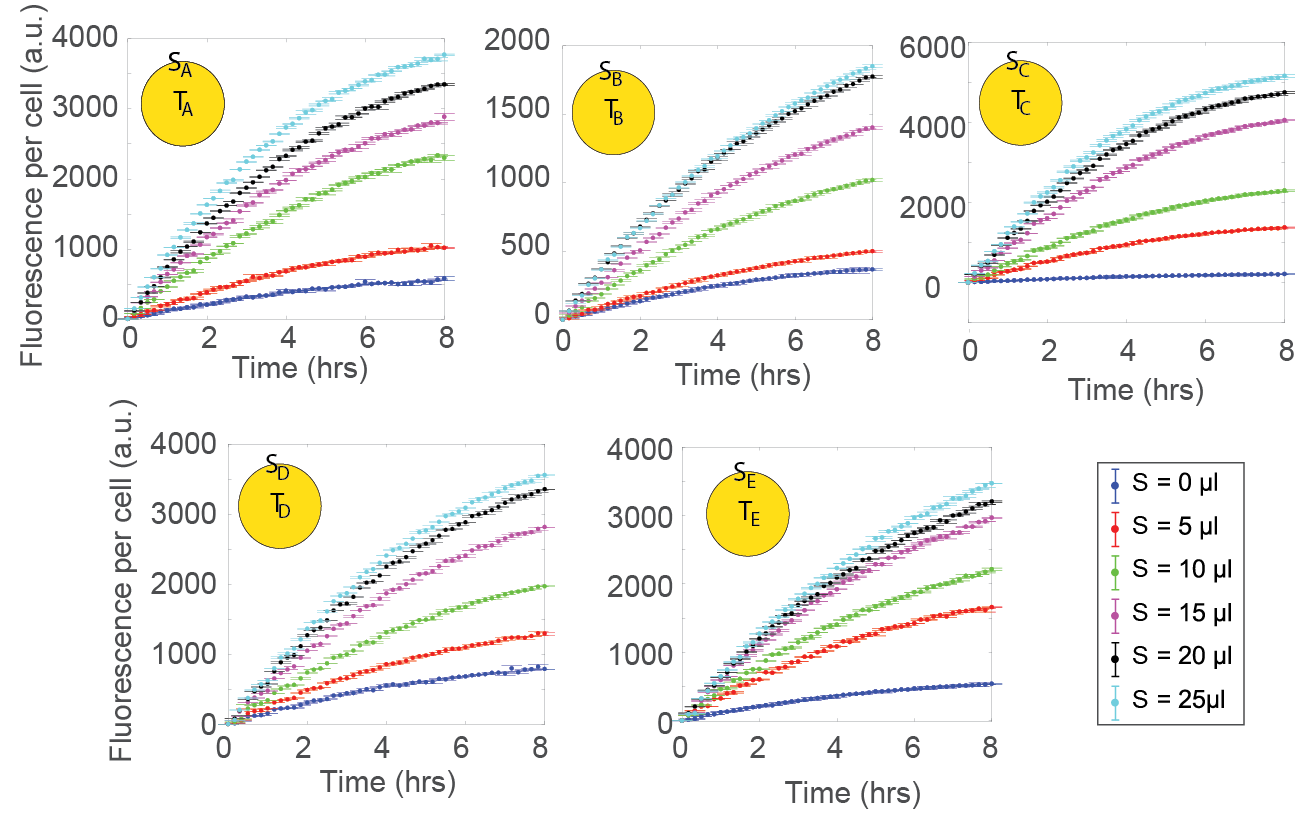
Fig A: Fluorescence per cell vs time for the testers when mixed with their cognate supernatants (indicated by the yellow circles). For each case, the multiple lines represent the amount of supernatant (S) used. Errorbars represent the standard error from two replicates.


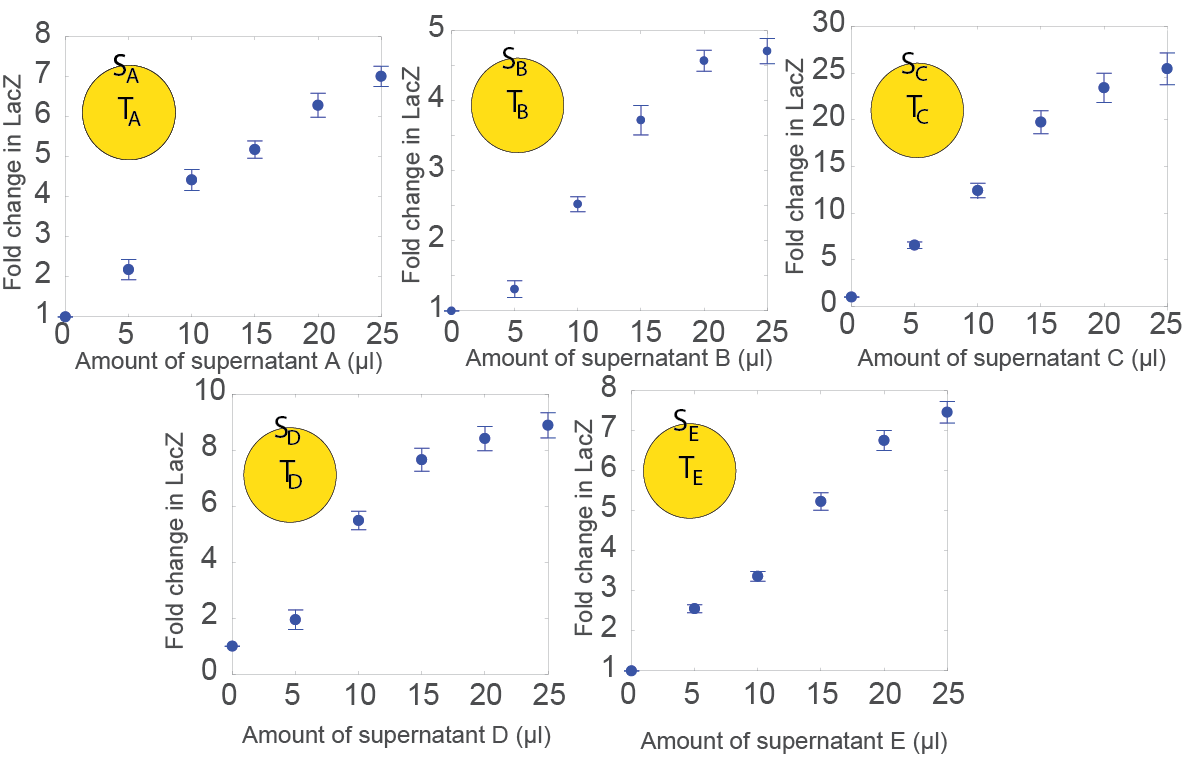


Fig B: The fold changes in the LacZ expression of each tester A, B, C, D and E respectively, when mixed with its cognate producer supernatant. The LacZ expression is based off of the rate of fluorescence increase which is the gradient of the fluorescent response between 0-5 hrs. Error bars represent the standard error from replicates obtained from over 4 days separately.


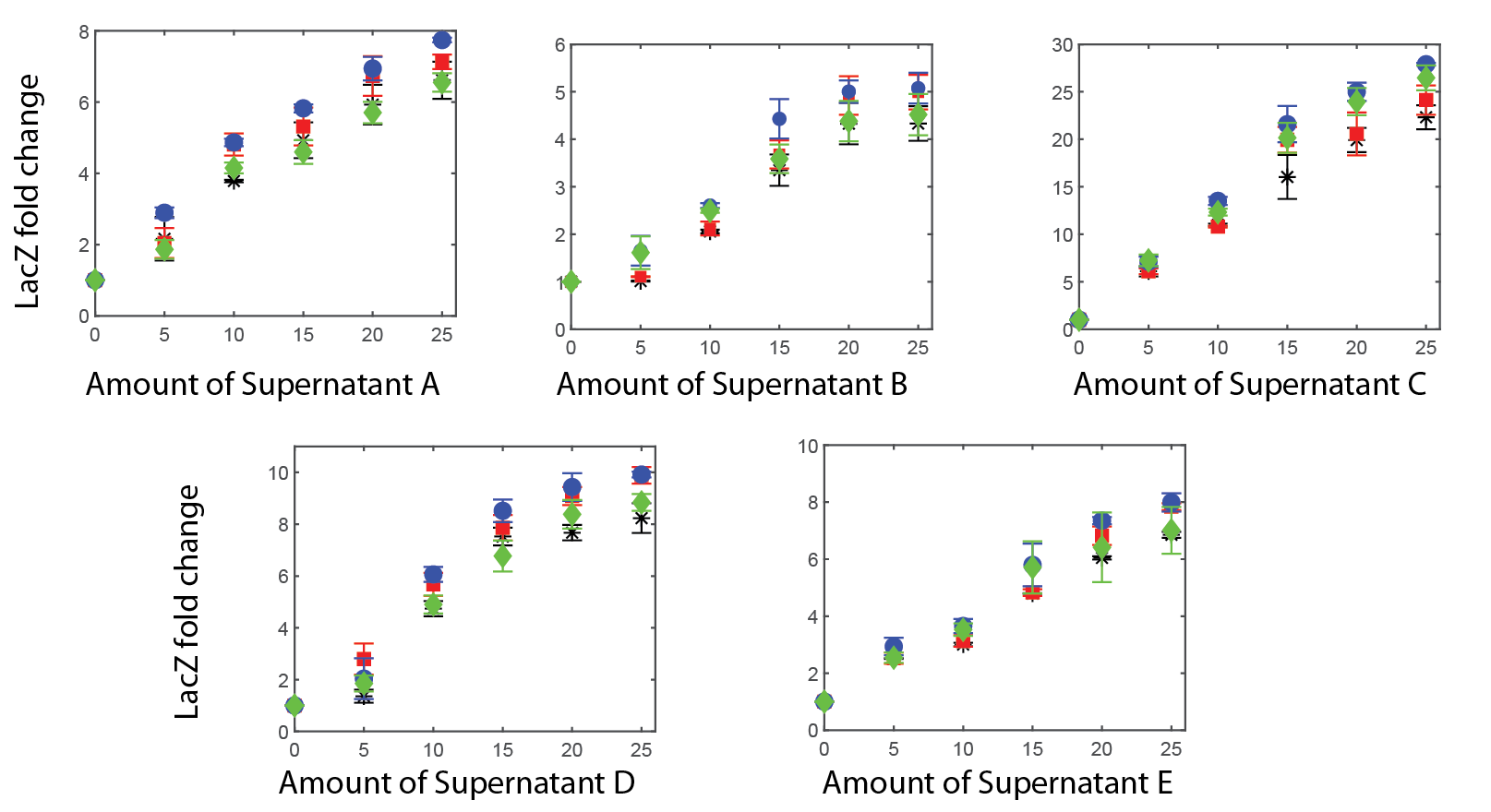


Fig C: The fold changes in the LacZ expression of each tester A, B, C, D and E respectively, when mixed with its cognate producer supernatant. Each symbol replicates a trial that was run on a separate day. The response of the testers were consistent throughout the dates. The dates were spread out during three weeks. The error bars indicated are the standard error from 2 sets of replicate run on the same day.


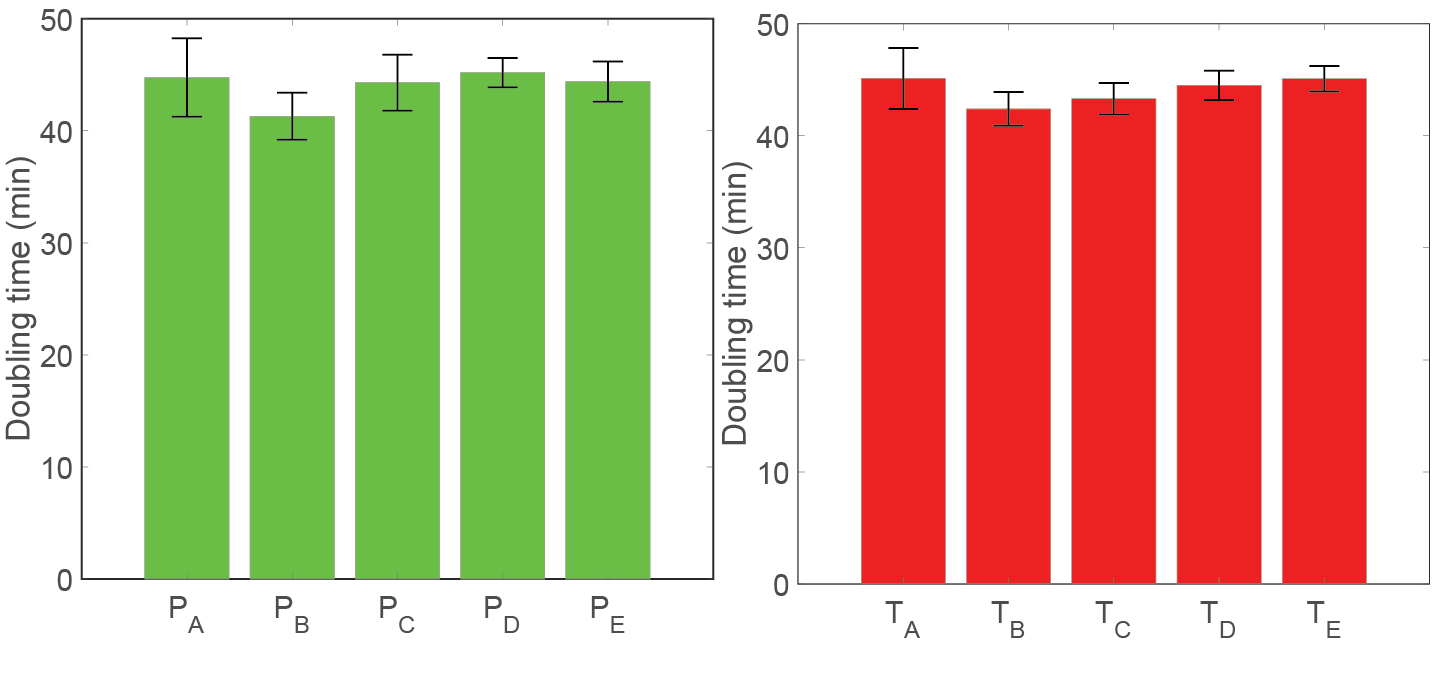


Fig D: Doubling times for the *B. subtilis* producers and testers, see methods for further details. Growth was measured by counting the colony forming units with respect to time in agar plates containing competence media. A linear fit was considered for the data points between 2hrs-6hrs, using the least squared fitting method and the gradient was extracted to obtain the growth rates. The estimated growth rates (in 1/hr) are, $\mu_{P_{A}}=0.920$_,_ $\mu_{P_{B}}=1.009$_,_ $\mu_{P_{C}}=0.924$_,_ $\mu_{P_{D}}=0.918$_,_ $\mu_{P_{E}}=0.937$_,_ $\mu_{T_{A}}=0.916$_,_ $\mu_{T_{B}}=0.990$_,_$\mu_{T_{C}}=0.943$_,_ $\mu_{T_{D}}=0.926$_,_ $\mu_{T_{E}}=0.916$_._ Errorbars are the standard deviation from three sets of trials.

| Parameter | Value | Obtained from |
| --- | --- | --- |
| $\mu_{i}$ | 0.916-1.009 hrs^-1^ | Calculated in this study, see Fig. S4 |
| $s$ | 10^9^ cells ml^-1^ | Silva *et al.* (2017) [36] |
| $\rho_{L}$ | 3.180 x 10^-7^ nM cell^−1^ hrs^−1^ | Haustenne *et al.* (2015) [40] |
| $\gamma_{L}$ | 0.600 hrs^-1^ | Santilla *et al.* (2008) [39] |
| $\rho_{c_{i}}$ | 3.180 x 10^-7^ nM cell^−1^ hrs^−1^ | Haustenne *et al.* (2015) [40] |
| $\gamma_{c_{i}}$ | 0.420 hrs^-1^ | Santilla *et al.* (2008) [39] |
| $m$ | 3 | Santilla *et al.* (2008) [39] |

Table A: Parameter values used in the study for the modeling.


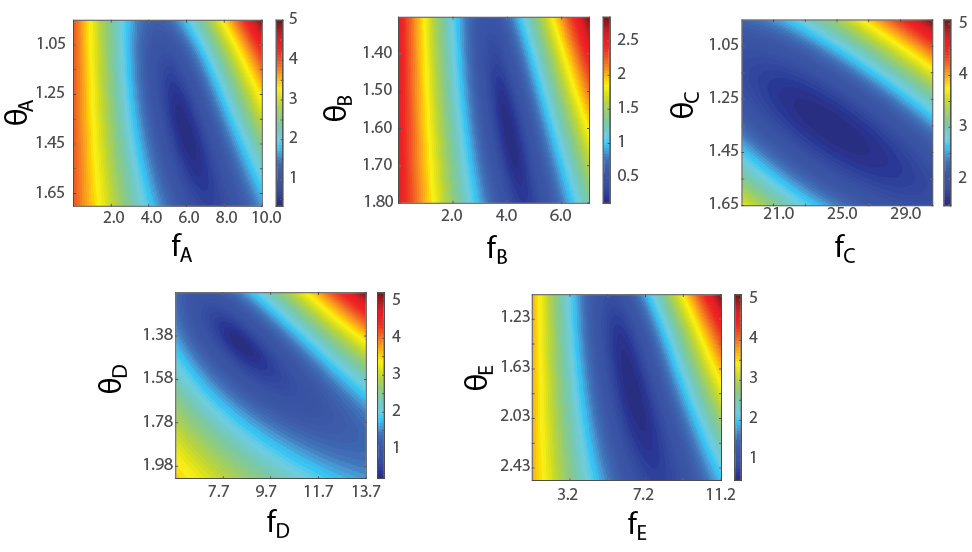


Fig E: The root mean squared error (indicated with the color intensity) was minimized for changing values of θ and f. The θ and f values that gave the minimum root mean squared error were averaged to obtain the values used in the simulations.


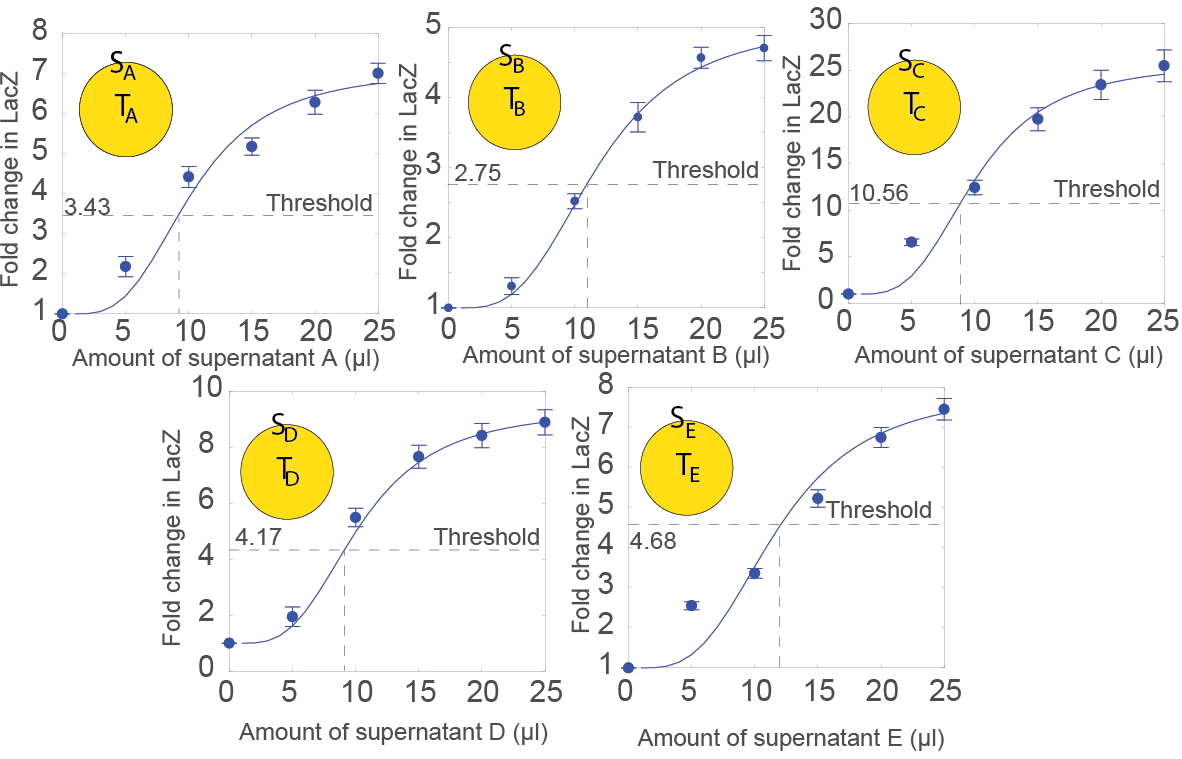


Fig F: The best fit curves for f and θ. These curves, obtained from simulations, were fit by minimizing the root mean square error for changing values of both f and θ. Once the θ was extracted, the fold change LacZ for that θ was found from the fitted curve. The fold change at θ was defined as the threshold needed for QS activation.


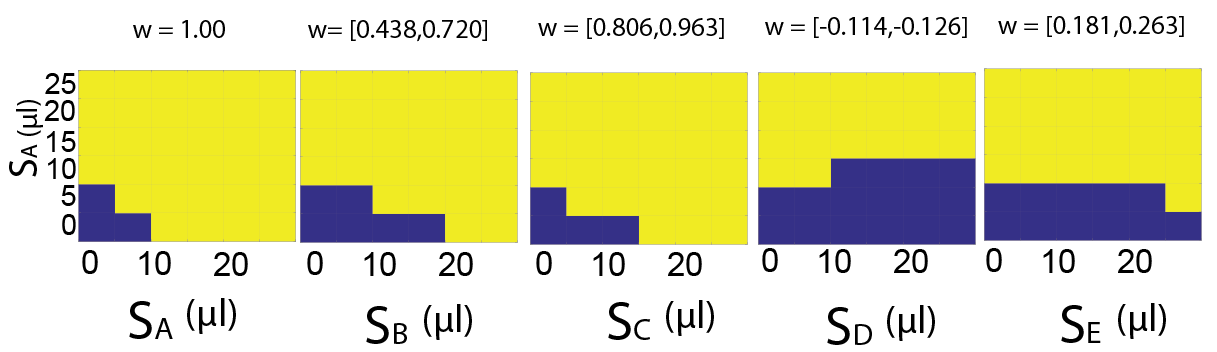


Fig G: The quorum sensing activation landscape for the tester A when mixed with the supernatant of producer A and the supernatant of producer A, B, C, D and E respectively (from left to right). A blue square represents quorum sensing activation off and a yellow square represents quorum sensing activation on. The weight values listed on top represent the range of values that generated the same activation landscape.


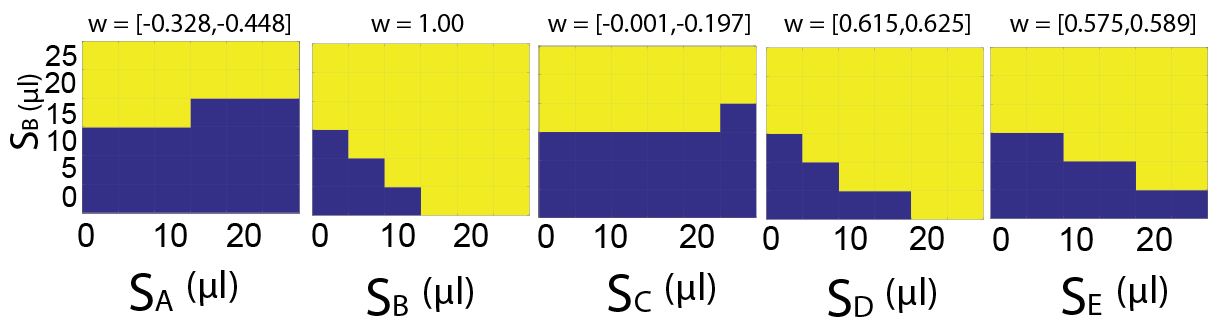


Fig H: The quorum sensing activation landscape for the tester B when mixed with the supernatant of producer B and the supernatant of producer A, B, C, D and E respectively (from left to right). A blue square represents quorum sensing activation off and a yellow square represents quorum sensing activation on. The weight values listed on top represent the range of values that generated the same activation landscape.


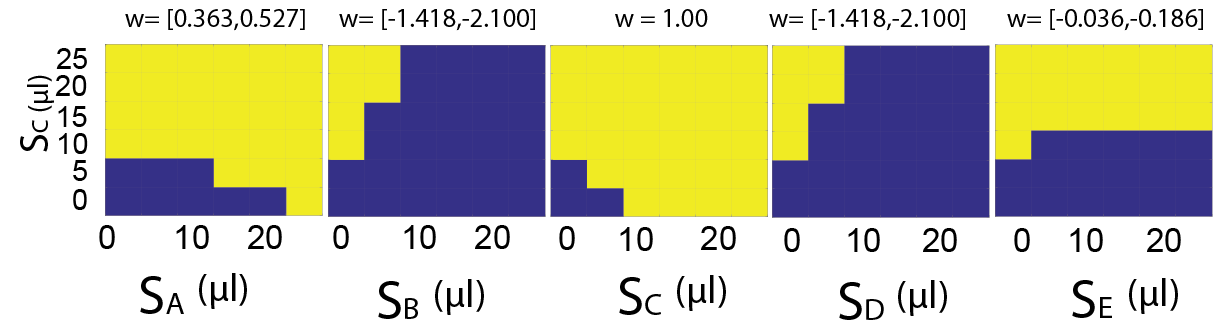


Fig I: The quorum sensing activation landscape for the tester C when mixed with the supernatant of producer C and the supernatant of producer A, B, C, D and E respectively (from left to right). A blue square represents quorum sensing activation off and a yellow square represents quorum sensing activation on. The weight values listed on top represent the range of values that generated the same activation landscape.


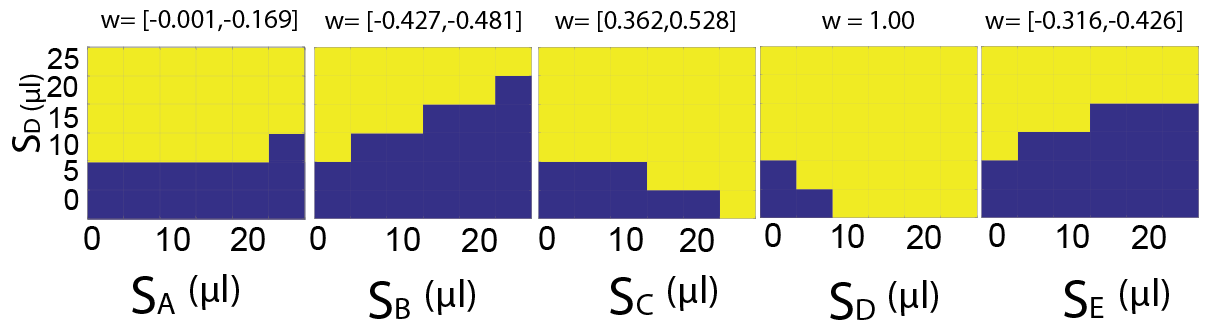


Fig J: The quorum sensing activation landscape for the tester D when mixed with the supernatant of producer D and the supernatant of producer A, B, C, D and E respectively (from left to right). A blue square represents quorum sensing activation off and a yellow square represents quorum sensing activation on. The weight values listed on top represent the range of values that generated the same activation landscape.


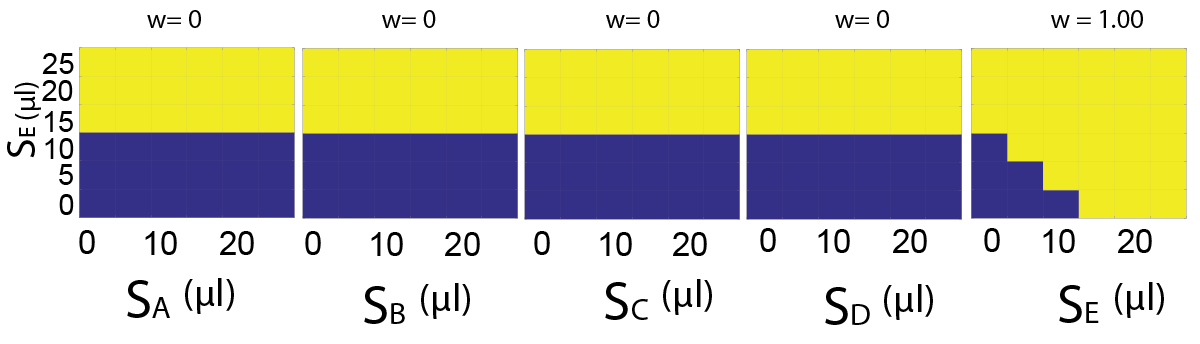
_­_

Fig K: The quorum sensing activation landscape for the tester E when mixed with the supernatant of producer E and the supernatant of producer A, B, C, D and E respectively (from left to right). A blue square represents quorum sensing activation off and a yellow square represents quorum sensing activation on. The weight values listed on top represent the range of values that generated the same activation landscape.


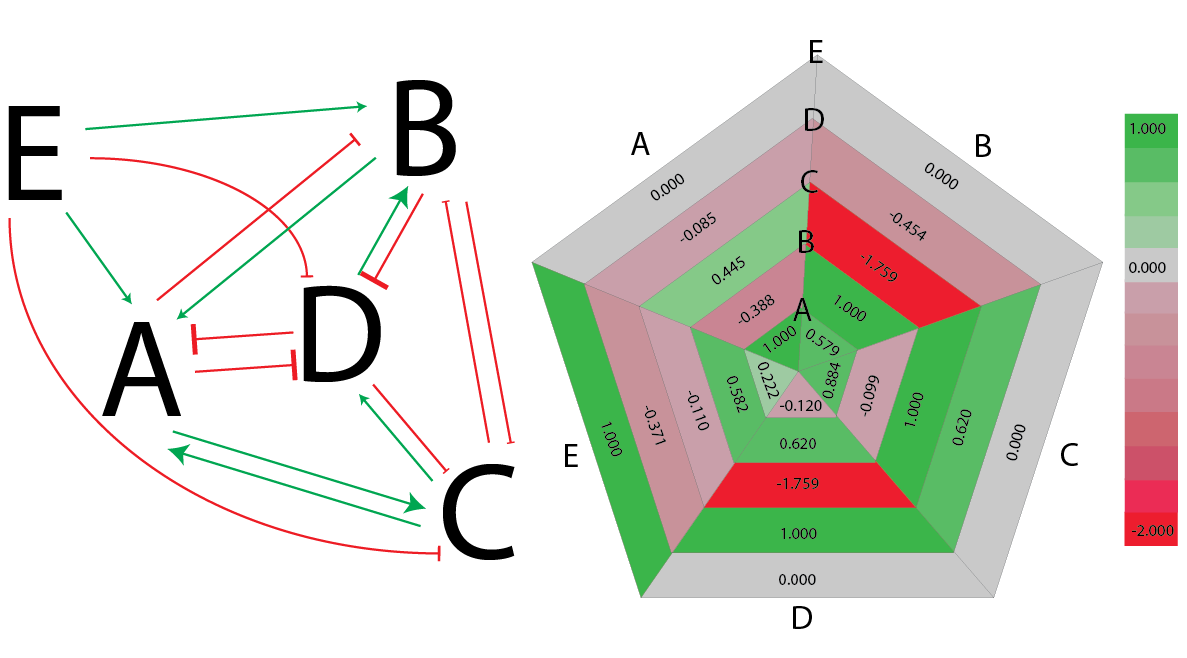


Fig L: The overall pairwise quorum sensing interaction map. In the left, the green arrow represents activation, the red flat head represents inhibition. On the right, we have elaborated the strength of each pairwise interaction. Each pentagram represents the strength of the pairwise interaction between a tester and all the supernatants. For instance, the smallest pentagram represents the response of T_A_. The colors represent the weight values. Green represents positive weights while red represents negative weights.


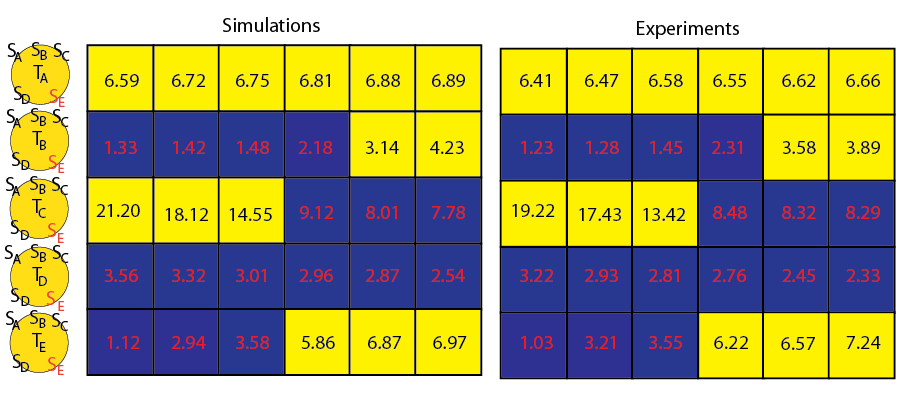


Fig M: LacZ fold change comparisons of the 5 supernatant case from simulations and experiments. Here the values indicated are the fold change in LacZ when increasing S_E_ from simulations (left) and experiments (right). Here QS on is represented by the color yellow and QS off is represented by the color blue. The threshold fold changes for QS activation of the strains A, B, C, D, E are 3.43, 2.75, 10.56, 4.17 and 4.68 respectively.


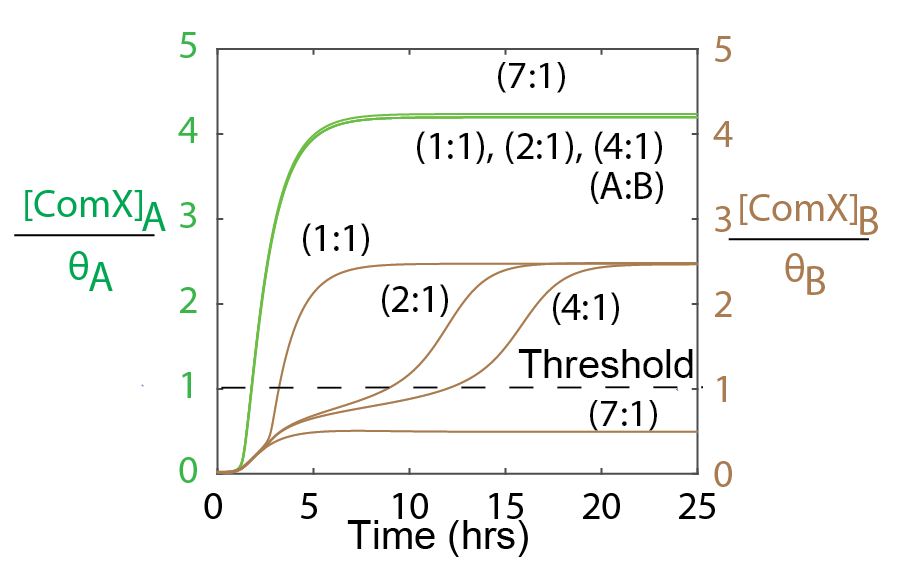


Fig N: Simulation results of the ComX concentration (normalized by Ɵ) over time for cocultures of P_A_ (green, left y-axis) and P_B_ (brown, right y-axis). Inoculation ratios (P_A_:P_B_) for the cocultures are noted in parentheses. For an inoculation ratio of 7:1, P_B_ will not activate QS.


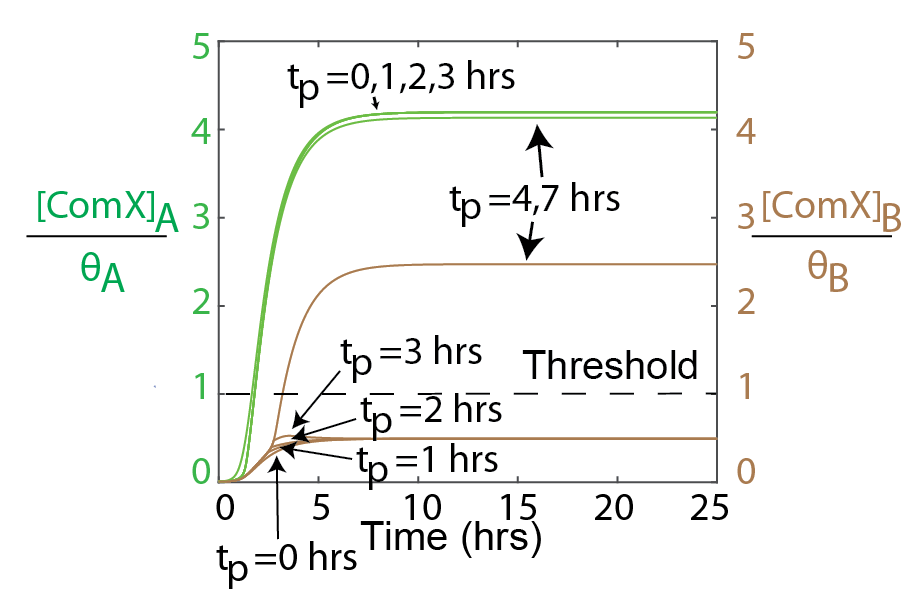


Fig O: Simulation results of the ComX concentration normalized by Ɵ over time for P_A_ (green, left y-axis) and P_B_ (brown, right y-axis) when they are mixed (P_A_: P_B_) at a ratio of 1:1. As observed in Fig. N, when the ratio was set to 1:1, both P_A_ and P_B_ activated QS. When S_C_ is added externally at a volume of 200 µl, the activation of P_B_ depends on the time at which S_C_ was introduced (t_p_ = time of perturbation) to the mixture. Since C will activate A, P_A_ activates QS for all t_p_.


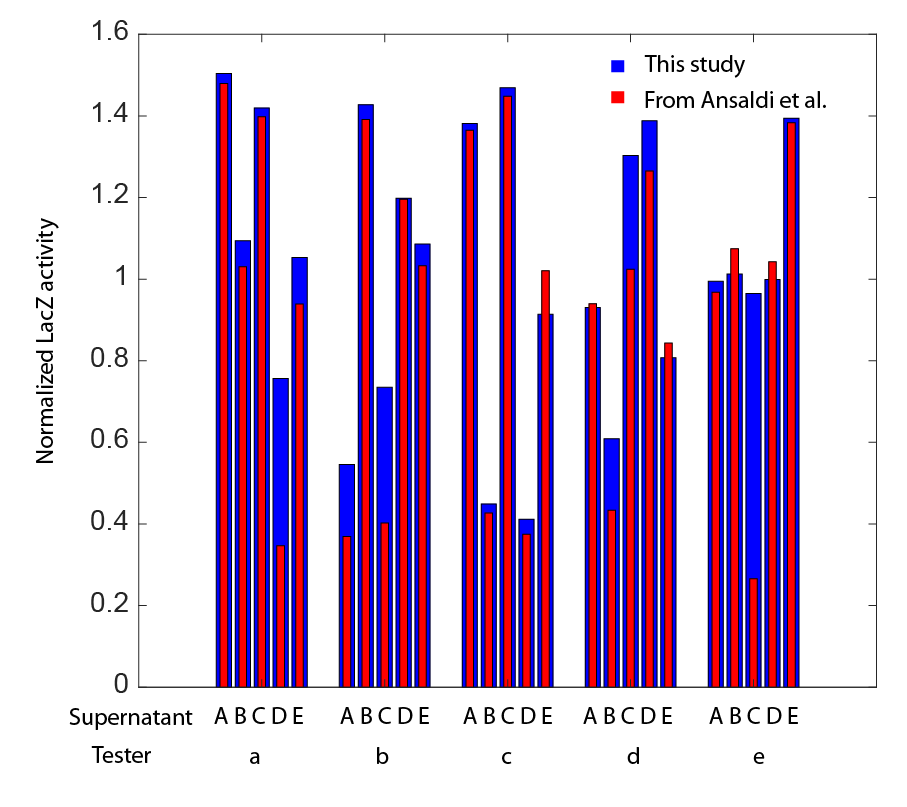


Figure P: The comparisons of the pairwise crosstalk of our results with Ansaldi *et al.* [9]. In this previous study, the LacZ activity of each tester was measured and crosstalk was tested by mixing the cognate ComX signal and a non-cognate ComX signal at a ratio of 1:1 obtained from purifying supernatants of *E. coli* producers. They tested if the LacZ expression was above or below the LacZ level when there was only the cognate ComX present. Here we compare these results with the LacZ measurements of the testers when the ComX signals were introduced at a ratio of 1:1. The normalized LacZ activity was the ratio between the LacZ activity of the pairwise combinations and the LacZ activity when only the cognate ComX signal is present. The ComX signal of our experiments were obtained from the *B. subtilis* producers as described from Tortosa *et al.* and as suggested in Ansaldi *et al.*, we observe similar levels of activity for most cases. We obtained the results of the previous study using the software plot digitizer. The blue bars represent the results from our study and the red bars represent the results from Ansaldi *et al.*

References:

1. Silva KP, Chellamuthu P, Boedicker JQ. Signal Destruction Tunes the Zone of Activation in Spatially Distributed Signaling Networks. Biophys J. Biophysical Society; 2017;112: 1037–1044. doi:10.1016/j.bpj.2017.01.010

2. Haustenne L, Bastin G, Hols P, Fontaine L. Modeling of the ComRS Signaling Pathway Reveals the Limiting Factors Controlling Competence in Streptococcus thermophilus. Front Microbiol. 2015;6: 1–20. doi:10.3389/fmicb.2015.01413

3. Santilla M. Bistable Behavior in a Model of the lac Operon in Escherichia coli with Variable Growth Rate. Biophys J. 2008;94: 2065–2081. doi:10.1529/biophysj.107.118026

4. Ansaldi M, Marolt D, Stebe T, Mandic-Mulec I, Dubnau D. Specific activation of the Bacillus quorum-sensing systems by isoprenylated pheromone variants. Mol Microbiol. 2002;44: 1561–1573. doi:10.1046/j.1365-2958.2002.02977.x

5. Tortosa P, Logsdon L, Kraigher B, Itoh Y, Mandic-Mulec I, Dubnau D. Specificity and genetic polymorphism of the Bacillus competence quorum-sensing system. J Bacteriol. 2001;183: 451–460. doi:10.1128/JB.183.2.451-460.2001
